# Analysis of patient repositioning accuracy in precision radiation therapy using automated image fusion

**DOI:** 10.1120/jacmp.v6i1.1998

**Published:** 2005-03-17

**Authors:** James L. Robar, Brenda G. Clark, Jason W. Schella, Chang Seon Kim

**Affiliations:** ^1^ Department of Medical Physics, Nova Scotia Cancer Centre and Department of Radiation Oncology Dalhousie University 5820 University Avenue Halifax Nova Scotia B3H 1V7 Canada; ^2^ Department of Medical Physics, BC Cancer Agency, Vancouver Centre and Department of Physics and Astronomy University of British Columbia 600 West 10th Avenue Vancouver British Columbia V5Z 4E6 Canada

**Keywords:** radiation therapy, immobilization, intensity‐modulated radiation therapy, stereotactic radiotherapy, stereotactic radiosurgery

## Abstract

This work describes a rapid and objective method of determining repositioning error during the course of precision radiation therapy using off‐line CT imaging and automated mutual‐information image fusion. The technique eliminates the variability associated with manual identification of anatomical landmarks by observers. A phantom study was conducted to quantify the accuracy of the image co‐registration‐based analysis itself. For CT voxel dimensions of $0.65\times 0.65\times 1.0\;{\text{mm}}^{3}$, the method is shown to detect translations with an accuracy of 0.5 mm in the anterior‐posterior and lateral dimensions and 0.8 mm in the superior‐inferior dimension. Phantom rotation in the coronal plane was detected to within 0.5° of expected values. The analysis has been applied to eight radiotherapy patients at two independent clinics, each immobilized by the same system for cranial stereotactic radiotherapy and CT‐imaged once per week over the five‐ to six‐week course of treatment. Among all patients, the ranges of translation in the anterior‐posterior, lateral, and superior‐inferior dimensions were −0.91mmto0.77mm,−0.66mm to1.02mm, and −2.24mm to3.47mm, respectively. Considering all patients and CT scans, the standard deviations of translation were 0.42 mm, 0.47 mm, and 1.36 mm in the anterior‐posterior, lateral, and superior‐inferior dimensions, respectively. The ranges of patient rotation about the superior‐inferior, left‐right, and anterior‐posterior axes were −2.84to2.62°,−1.74°to1.96°, and −1.78°to1.42°, respectively.

PACS numbers: 87.53.‐j, 87.53.Kn, 87.53.Ly, 87.53.Xd

## I. INTRODUCTION

Fractionated stereotactic radiotherapy (SRT) is a widely‐used treatment technique requiring stringent control over the spatial accuracy of the radiation dose delivered. Compared to single‐fraction stereotactic radiosurgery, SRT involves similar spatial tolerances on target localization and isocentric stability. However, the fractionated dose regimen of SRT usually necessitates the use of a relocatable immobilization system such as a rigid thermoplastic mask. The accuracy of inter‐fraction patient repositioning achievable with such a system is essential to accurate treatment delivery and therefore should be established quantitatively.

The accuracy of inter‐fractional patient repositioning has been investigated for various immobilization systems by acquiring sequential orthogonal radiographs or portal images.^(^ ^1^ ^–^ ^12^ ^)^ This approach usually involves the acquisition of anterior‐posterior and lateral projection radiographs of the patient anatomy, determination of locations of anatomical landmarks in relation to a fixed reference point (e.g., in the stereotactic coordinate system), and subsequent comparison of these locations to those from reference images (e.g., radiographic images acquired at the time of patient simulation or digitally‐reconstructed radiographs.) This method is straightforward and may be performed on a radiotherapy simulator or LINAC by radiation therapists throughout the course of treatment. However, the identification or superimposition of anatomical landmarks is required by skilled observers, and the identification process itself may introduce variability, particularly in megavoltage portal images. If rigid immobilization is used, the magnitude of actual repositioning errors may be on the order of 3 mm or less^(3)^ ^,^ ^(9)^ ^,^ ^(13)^ ^–^ ^(15)^; thus the uncertainty in the observation may represent a significant proportion of the error itself.^(15)^ Another shortcoming of this approach is that the location of the gross tumor volume itself is not observed directly, and shifts of the target volume are determined only in relation to clearly‐visualized structures,^(3)^ ^,^ ^(6)^ ^,^ ^(8)^ ^,^ ^(9)^ ^,^ ^(12)^ such as bony anatomy.

Other investigators have quantified patient repositioning accuracy based on three‐dimensional image data acquired by performing verification CT scans prior to or during the course of treatment.^(^ ^13^ ^–^ ^17^ ^)^ For cranial stereotactic treatment, the shift in the isocenter location was estimated by comparing the coordinates of manually identified landmarks such as gyri, ventricles, sinuses, and bony structures between the initial and verification image sets.^(13)^ ^,^ ^(14)^ ^,^ ^(16)^ Studies using this approach have been performed for paraspinal radiosurgery, wherein the patient repositioning errors were quantified through identification of bony landmarks,^(15)^ ^,^ ^(18)^ calcifications, or metallic implants.^(18)^ Lohr et al.^(15)^ have estimated the uncertainty associated with landmark identification to be ±1mm, with an additional ±1mm inherent in the measurement process itself, primarily determined by the CT voxel size.

In this work, we describe an automated and reproducible CT co‐registration‐based technique to quantify patient repositioning accuracy of a commercially available SRT immobilization system in precision radiotherapy. It is anticipated that the advantages of this method over other approaches are twofold: (1) to improve the speed and accuracy of the repositioning analysis by eliminating the need for manual identification of anatomical landmarks and (2) to allow full characterization of the translation and rotation of the cranium. This paper describes the methodology of an analysis that should be applicable using most commercial treatment‐planning systems with mutual‐information fusion capability. The accuracy and precision of the method are estimated based on the results of a controlled anthropomorphic phantom study, and the repositioning results of eight SRT patients from two independent clinics are presented.

## II. METHODS

### A. Stereotactic radiotherapy mask system

**Figure 1:**
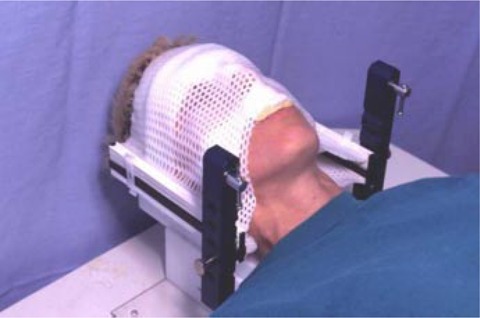
The patient immobilization system used at both institutions (BrainLAB cranial SRT mask system, BrainLAB AG). Anterior and occipital rigid thermoplastic halves of the mask are clipped to black carbon fiber posts.

The same commercial stereotactic radiotherapy mask system (BrainLAB mask system for SRT, BrainLAB AG) was used by both the BC Cancer Agency, Vancouver Centre and the Nova Scotia Cancer Centre, Halifax, Canada. This system consists of a “U”‐shaped frame to which two carbon fiber posts are attached to extend superiorly (Fig. 1). Separate anterior and posterior halves of the perforated thermoplastic mask are fitted close to the patient's head; plastic clips attached to the carbon fiber posts secure the masks. Adjacent to the patient's skin, the anterior half of the mask is reinforced with two solid thermoplastic strips: one above the eyes and the other below the nose. A bite‐block is attached to the more inferior of these strips. A solid mass of malleable thermoplastic is formed to the nasal bridge and attached to the mask. Following fabrication, the patient remains in the mask for 30 min to minimize the possible effects of thermoplastic shrinkage during cooling. Throughout the course of treatment, the separation between the anterior and posterior halves of the mask is set by the use of plastic spacers on the surface of the carbon fiber posts. This separation can be modified in 1.0 mm increments to accommodate changes in patient weight or swelling, although this is a rare occurrence in our treatment centers. This particular stereotactic mask system has been described by Alheit et al.,^(9)^ Willner et al.,^(13)^ and Wong et al.^(14)^

### B. CT simulation, planning, and follow‐up CT imaging

Eight patients were selected at random for CT verification during the course of their SRT treatment (five from the BC Cancer Agency and three from the Nova Scotia Cancer Centre). At both centers, all CT imaging was performed using Picker PQ‐2000 CT scanners (Philips Medical Systems, Andover MA). For the CT simulation prior to treatment planning, the CT localizer box is mounted to the U‐shaped frame via three ball‐attachments. CT scanning was done in spiral mode using a pitch of 1.5, 1.0 mm reconstructed slice‐thickness, no slice overlap in the vicinity of the lesion to be treated, and a pixel size of 0.65 mm. Imaging was performed to include the superior aspect of the head and the inferior end of the fiducial region of the localizer box. After importing a CT set into the planning system (BrainSCAN, BrainLAB AG), stereotactic localization was performed by the software by identifying the location of each of six localizer fiducials on the outside surfaces of the left, right, and anterior walls of the localizer box. Localization thus establishes the three‐dimensional stereotactic coordinate system for treatment planning and delivery. Following normal structure and tumor volume contouring, SRT treatment plans were established, and the patients began treatment. Over the course of the five‐to six‐week duration of treatment, each patient was CT‐scanned once per week, for a total of 44 CT scans (four patients underwent 5 scans, and four patients underwent 6 scans, including the initial CT simulation and verification scans). For the verification CT studies, the setup within the SRT mask system and the CT protocol replicated those used for the initial simulation. Precise alignment of the localizer box relative to the CT scanner coordinate system (i.e., as indicated by the CT lasers) is not critical, since the planning software transforms the CT images to the stereotactic coordinate system.

### C. Image co‐registration‐based analysis

For each patient, the goal of the image co‐registration‐based analysis is to determine the translation and rotation of each verification CT scan relative to the initial simulation CT scan. While the method takes advantage of automated image fusion within the treatment‐planning system, the planning system does not provide the translation matrices to the user explicitly; therefore, as outlined below, the procedure involves the acquisition of the coordinates of corresponding points within the verification CT set prior to, and following the image fusion.

**Figure 2:**
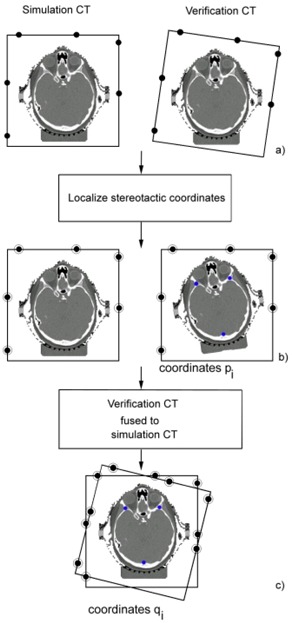
The methodology used for the co‐registration‐based analysis. (a) After importing the CT sets into the planning system, the stereotactic coordinate systems are not co‐registered and the anatomy is unfused. (b) Following fiducial localization, the stereotactic coordinate systems are matched, but (in the presence of repositioning error) the anatomy is not co‐registered. Three small reference contours are added, and the coordinates of the centers of these contours are obtained. (c) After the image fusion, the stereotactic coordinate systems are not co‐registered but the anatomy is fused, and the reference contours have undergone the same transformation as the verification CT. The new coordinates of the reference contours are recorded.

1. After importing both the simulation and verification CT sets into the planning system, the image sets are neither localized to stereotactic coordinates nor fused (Fig. 2(a).
2. The simulation CT set and all verification CT sets are localized automatically by the planning software through identification of the stereotactic fiducials (Fig. 2(b). This step spatially co‐registers the stereotactic coordinate systems of all CT sets, but in the event of patient repositioning error with respect to the localizer box, the anatomy within the localizer box will be spatially mismatched.
3. Three small, circular reference contours are drawn in the verification CT set (Fig. 2(b), right). The locations of these marks are well separated (over 10 cm apart) in three dimensions but do not necessarily correspond to particular anatomical features. While the absolute dimension of these contours is not critical, the diameter of each contour was set approximately equal to the CT pixel size. The planning system does not limit the pixel size in drawn contours to the pixel size of the underlying CT scan.
4. For each CT set, a temporary isocenter is placed within each circular reference contour. Because the planning system has the capability of placing the isocenter at the center of mass of any defined structure, the temporary isocenter is located at the center of the circular contour. This process facilitates acquisition of a set of prefusion coordinates, $p_{i}$, for all three landmarks with a precision of less than the pixel dimension.
5. The verification CT set is then fused to the simulation CT set using the automated mutual information‐based fusion algorithm included in the planning system. The fusion algorithm is a commercial implementation based on the approach of Thevenaz et al.^(19)^ and Studholme et al.^(20)^ and is normally used in the treatment‐planning process for fusion of CT, MRI, and/or PET image sets. The iterative algorithm assumes the anatomy to be a rigid body and determines six transformation parameters (three degrees of freedom for both translation and rotation) that maximize a similarity measure between spatially coincident voxel pairs. The fusion process typically requires less than one minute per CT set of approximately 150 slices and requires no user intervention. Following the image fusion, if patient repositioning error is present between the simulation and verification image sets, the stereotactic coordinate systems are no longer co‐registered (i.e., overlay of localizer bars exhibits mismatch), but the patient anatomy is spatially matched, as shown in Fig. 2(c). Since each circular reference contour is “attached” to an individual verification CT set, as the CT set is translated and rotated during the fusion process, the circular reference contours undergo the same transformation.
6. Step 4 is repeated to obtain postfusion coordinates, $q_{i}$, for the set of reference contours. The two point sets $p_{i}$ and $q_{i}$ are translated to the origin by subtracting their centroid coordinates, that is,
  $$\begin{array}{l} p\prime =p_{i}-p_{\text{centroid}}\\ q\prime =q_{i}-q_{\text{centroid},} \end{array}$$
  where $p_{\text{centroid}}$ and $q_{\text{centroid}}$ are the centroid coordinates of the three reference contours, before and after fusion, respectively. The translation matrix *T* is then determined by
  $$T=q_{\text{centroid}}-p_{\text{centroid}}$$

Positive values indicate translations in the anterior, left, and superior directions. Next, the least‐squares solution to the system of equations

p′R=q′

is solved (MATLAB, The Mathworks Inc.) for the combined pitch, roll, and yaw rotation matrix *R*, where

$$R=\left[\begin{array}{ccc} \text{cos}\;\alpha \;\text{cos}\;\beta & \text{sin}\;\alpha \;\text{cos}\;\gamma +\text{cos}\;\alpha \;\text{sin}\;\beta \;\text{sin}\;\gamma & \text{sin}\;\alpha \;\text{sin}\;\gamma -\text{cos}\;\alpha \;\text{sin}\;\beta \;\text{cos}\;\gamma \\ -\text{sin}\;\alpha \;\text{cos}\;\beta & \text{cos}\;\alpha \;\text{cos}\;\gamma -\text{sin}\;\alpha \;\text{sin}\;\beta \;\text{sin}\;\gamma & \text{cos}\;\alpha \;\text{sin}\;\gamma +\text{sin}\;\alpha \;\text{sin}\;\beta \;\text{cos}\;\gamma \\ \text{sin}\;\beta & -\text{cos}\;\beta \;\text{sin}\;\gamma & \text{cos}\;\beta \;\text{cos}\;\gamma \end{array}\right]$$

Angles α, β, and γ represent counter clockwise rotations about the superior‐inferior, left‐right, and anterior‐posterior axes, respectively, when viewed from a point on a positive axis and toward the origin. Having determined the rotation matrix *R*, these angles are calculated as follows:

$$\begin{array}{l} \alpha ={\text{tan}}^{-1}\left(\frac{-r_{21}}{r_{11}}\right)\\ \beta ={\text{tan}}^{-1}\left(\frac{r_{31}}{\sqrt{r_{32}^{2}+r_{33}^{2}}}\right)\\ \gamma ={\text{tan}}^{-1}\left(\frac{-r_{32}}{r_{33}}\right), \end{array}$$

where the first and second subscripts of *r* indicate the row and column indices of the matrix *R*, respectively.

### D. Phantom study: Validation of image co‐registration‐based analysis

**Figure 3:**
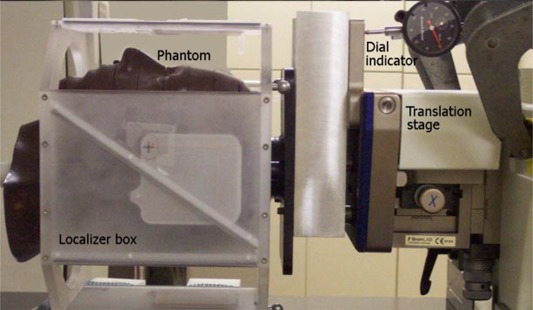
Physical setup of the phantom on the CT couch showing placement of the head phantom inside of the stereotactic localizer box and dial indicator.

Although the analysis method described above does not rely on subjective identification of landmarks by the user, its accuracy will be dictated by that of the fusion algorithm itself. Therefore, the accuracy of the complete transformation matrix of the fusion algorithm must be established. A separate study was carried out in which known translations and rotations in the coronal plane of an anthropomorphic head phantom were introduced in separate CT sets relative to a baseline CT set. The physical setup for this test is shown in Fig. 3. The superior end of the head phantom (Rando Phantom, The Phantom Laboratory, NY) was bolted securely to a surface plate, which in turn was mounted to a three‐dimensional translation stage allowing fine adjustments (the stereotactic couch mount was adapted to sit securely on the surface of the CT couch). The localizer box was fixed to the CT couch to allow translation of the phantom within the localizer box without movement of the stereotactic coordinate system, as shown in the figure. Multiple dial indicators were used to verify that the translation stage moved parallel to the axes of the localizer box. The same dial indicators were also used to measure the translation of the phantom in three dimensions with a precision of ±0.03mm. A baseline CT scan of the phantom was performed with the phantom located at a set position. Next, for each dimension separately, individual scans were done with the phantom offset by 0.25 mm, 0.50 mm, 1.00 mm, 2.00 mm, and 8.00 mm. Translations toward the posterior, left, and inferior were introduced separately for each scan; thus 18 CT sets were obtained (one baseline and five offsets per dimension).

Since the apparatus did not readily allow for rotation of the phantom itself, rotation was introduced relative to the stereotactic coordinate system instead by rotating the surrounding localizer box about the anterior‐posterior axis of the phantom. In doing so, the phantom remained stationary with its superior‐inferior axis aligned with the longitudinal axis of the CT gantry. To measure rotations introduced in this manner, the localizer box was placed on a plastic platform permitting rotations in a coronal plane (i.e., for adjustment of the angle γ as described above) about the center of the stereotactic coordinate system. A printed template was attached to the top of the localizer box showing the angle between the superior‐inferior axis of the localizer box and the longitudinal axis of the CT simulator, whereby the latter was indicated by an overlay of the sagittal CT simulator laser on the template. In this fashion, small rotations could be introduced with a precision of approximately ±0.3°. Relative to the baseline scan (with γ=0.0°), pure rotations were introduced at γ=−2.0° and γ=−5.0° in separate scans. Finally, to simulate a more realistic case, a rotation of γ=−5.0° was first introduced, followed by with offsets of 3.00 mm to the anterior and 4.00 mm to the right.

The CT imaging of the phantom was performed using the same protocol as used for patients. The repositioning analysis was conducted exactly as outlined above for patient CT sets. The phantom contains internal structures (bony anatomy and air cavities) and therefore provides suitable datasets for the mutual‐information fusion algorithm.

In summary, this procedure yields a set of “actual” translations and/or rotations with a precision of ±0.03mm and ±0.3°, respectively, spanning the range that would be expected in the context of patient immobilization, and a corresponding set of “detected” translations and/or rotations, as extracted by our method involving the image co‐registration‐based analysis.

## III. RESULTS

### A. Phantom study: Validation of image co‐registration‐based analysis

Table 1 compares the actual and detected translations and/or rotations for the various CT sets acquired for the phantom study. From the translation‐only studies, in the anterior‐posterior and lateral dimensions, the discrepancy between the detected and actual translations was less than 0.5 mm, and less than 0.35 mm averaged among all CT sets. For the superior‐inferior dimension, the discrepancy was 0.75 mm maximally and 0.60 mm on average. For all dimensions, the magnitude of the discrepancy does not appear to be correlated with the magnitude of the actual translation introduced. For dimensions in which translation was not actually introduced, the detected translation was below 0.3 mm among individual measurements and below 0.12 mm on average. For these studies, the detected rotations were close to zero as expected, within a range of −0.07° and 0.07°, and with mean values below 0.04°, among all phantom verification CT studies.

**Table 1:**
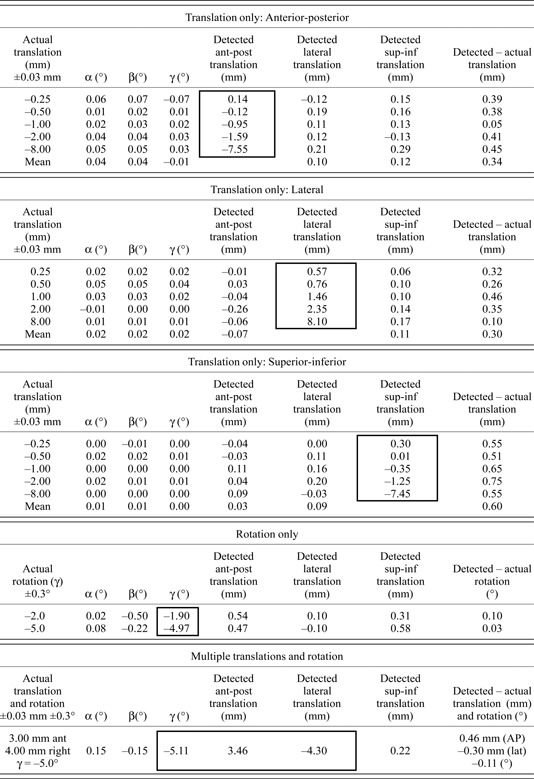
Phantom study results, comparing actual and detected translations and rotations. Outlined regions show detected results expected to be nonzero.

While the experiments described above aim to establish the accuracy of the co‐registration‐based analysis, it was also necessary to estimate the precision of the process. Therefore, the same shift of 2.0 mm was introduced five times at the CT scanner for each dimension, and the standard deviation σ of the detected translations was calculated. This was found to be 0.13 mm for both the anterior and posterior dimensions, and 0.09 mm for the superior‐inferior dimension.

Rotations introduced in the coronal plane were detected with discrepancies in γ below 0.1°. It should be noted that this is smaller than the estimated precision of physical rotation of the localizer box. Detected values of α and β differed from zero by −0.5°to0.15°. For the study involving rotation as well as translation in two dimensions, the actual translations were detected within 0.46 mm, and the rotation was detected within −0.11°.

### B. Patient repositioning accuracy

**Figure 4:**
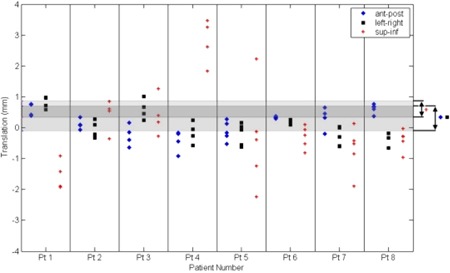
Translational repositioning errors for the cranium, for eight patients. Each symbol represents a single detected shift in the anterior‐posterior (♦), lateral (■), or superior‐inferior (+) dimensions. Note that several points overlap in this figure. Shaded bands indicate regions of nonsignificance for the anterior‐posterior and lateral dimensions (lower band) and superior‐inferior dimension (upper band). These bands are centered upon the mean discrepancy between the detected and actual displacements from the phantom study and have widths of ±3 standard deviations.

The detected translations of the cranium for all patients are plotted in Fig. 4. Patients 1 to 5 were treated at the BC Cancer Agency, and patients 6 to 8 were treated at the Nova Scotia Cancer Centre. Among all patients, the ranges of translation in the anterior‐posterior, lateral, and superior‐inferior dimensions are −0.91mm to0.77mm, −0.66mm to1.02mm, and −2.24mm to3.47mm, respectively. Among all patients and verification CT sets, the mean translations were 0.16 mm, 0.04 mm, and −0.02mm, respectively, indicating that, overall, no significant systematic bias was present in patient positioning between the initial planning and subsequent verification CT imaging. Patient positioning variability is indicated by the standard deviations of translation, which were 0.42 mm, 0.47 mm, and 1.36 mm, respectively, in anterior‐posterior, lateral, and superior‐inferior dimensions. The shaded bands in Fig. 4 indicate regions of nonsignificance, as estimated from the phantom study. These bands are centered upon the dimension‐specific mean of the discrepancy between actual and detected translations (which was the same to within 0.04 mm for the anterior‐posterior and lateral dimensions). The widths of the bands represent ±3σ, where σ was determined from repeated trials as described above. For the anterior‐posterior, lateral, and superior‐inferior dimensions, 22%, 50%, and 92% of measured translations lie outside of these bands, respectively.

**Figure 5:**
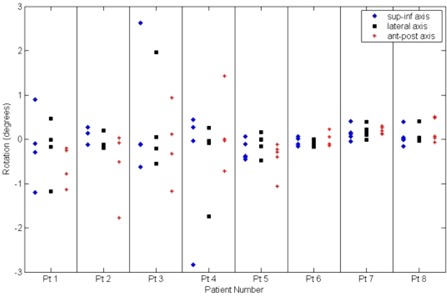
Rotational repositioning errors for the cranium, for eight patients, about the superior‐inferior (♦), lateral (■), and anterior‐posterior (+) axes.

The detected rotations for the eight patients are shown in Fig. 5. The ranges of rotation about the superior‐inferior, left‐right, and anterior‐posterior axes are −2.84°to2.62°, −1.74°to1.96°, and −1.78°to1.42°, respectively. Among all patients and CT sets, the mean values are −0.05°, −0.04°, and −0.14° for the three dimensions, respectively, and the standard deviations are 0.74°, 0.53°, and 0.59°, respectively.

## IV. DISCUSSION

Use of mutual‐information image fusion for quantification of patient repositioning accuracy offers several advantages. First, the method is sufficiently accurate and precise, as demonstrated by the phantom study described above. To put the accuracy of the method into context, the discrepancies between actual and detected translations (i.e., 0.5 mm for anterior‐posterior and lateral dimensions, and 0.8 mm for the superior‐inferior dimension, maximally) are, in fact, smaller than the voxel dimensions of the CT sets used. Compared to other image data used in the treatment‐planning process, these discrepancies are similar in magnitude to the voxel size of the magnetic resonance images (0.5mm×0.5mm×1.0mm) used routinely in our clinic as the primary reference for target volume and normal structure contouring. The discrepancies are also smaller than the finest grid size (1.0 mm) of dose calculations performed in our treatment‐planning system. In addition to being accurate, the method is also rapid, requiring several minutes for a single verification CT set. However, the process would be streamlined further by planning systems that provide the transformation matrices to the user; this would eliminate the need to acquire reference contour coordinates and to solve for the translation and rotation separately.

While this co‐registration‐based approach works well for the cranium, a clear limitation of the method is that the fusion is a rigid‐body affine transformation, and does not address distortion between the planning and verification image sets, for example, caused by anatomical changes between the planning and verification CT sessions. This may become an important issue, for example, for radiation therapy of the head and neck, in which significant anatomical changes can occur during the initial phase of treatment with weight loss and/or tumor volume shrinkage.

There are several necessary technical prerequisites for this approach. First, as seen in the results of the patient repositioning study, repositioning translational errors may be very small, especially for rigid immobilization systems. For example, in this work, it was seen that only 22% and 50% of detected translations occurred outside of the non‐significant bands of Fig. 4, for the anterior‐posterior and lateral dimensions, respectively. This emphasizes the requirement of sufficiently small voxel size in both the planning and verification CT sets. A second requirement is an automated and accurate voxel‐based fusion algorithm such as the mutual‐information approach described above. Such algorithms are well documented and are now offered by a number of commercial treatment‐planning systems. One would expect the accuracy of the fusion to be on the order of the voxel dimension since its primary function includes co‐registration of image sets prior to structure contouring, but the accuracy may be evaluated using phantom tests. It should be noted that the advantages of this method arise specifically from the mutual information algorithm itself, and that alternative fusion techniques involving operator intervention would not remove observer variability from the analysis. Moreover, algorithms requiring significant operator intervention would also make CT‐based assessment of repositioning error less attractive due to the time required.

The results of the patient study indicate that the BrainLAB SRT mask system provides reasonable inter‐fraction patient repositioning accuracy, exhibiting translational standard deviations of 0.42 mm, 0.47 mm, and 1.36 mm, respectively, in anterior‐posterior, lateral, and superior‐inferior dimensions. These values are within 1.5 mm of values of σ from other studies^(9)^ ^,^ ^(13)^ of the same mask system; however, direct comparison is limited by differences in imaging and analysis methods. Our work shows that repositioning is generally worse in the superior‐inferior dimension compared to the anterior‐posterior and lateral dimensions. The specific reason for this is not isolated by our data; however, we suggest that this may arise from the fact that while the patient position is set anteriorly‐posteriorly and laterally by rigid, continuous thermoplastic surfaces, in the superior‐inferior dimension, the mask is open at the top of the head and does not extend below the mandible. Although a bite block is used as a guide to setting the superior‐inferior position of the patient, it has been our experience that it does not contribute significantly to repositioning accuracy.

In summary, we have found that the mutual‐information fusion approach facilitates a rapid and objective assessment of repositioning accuracy in three scenarios:

1. characterization of the immobilization system accuracy during initial use and testing;
2. quality assurance and monitoring of a specific patient throughout the course of treatment using off‐line CT imaging;
3. assessment of systematic error caused by an incidental change of the physical setup. This third scenario may follow suspected wear and tear on the mask or other immobilizing components such as the bite block. The method is sufficiently straightforward and rapid to allow determination of the repositioning error in advance of the subsequent treatment fraction.

## V. CONCLUSION

The use of the automated mutual‐information image fusion functionality within the treatment‐planning system allows the assessment of patient repositioning accuracy. As demonstrated by a controlled phantom study, the fusion algorithm is capable of detecting translations with an accuracy less than the voxel dimension itself (in this study to within 0.5 mm, 0.5 mm, and 0.8 mm in anterior‐posterior, lateral, and superior‐inferior dimensions, respectively, for a voxel size of $1.0\times 0.65\times 0.65\;{\text{mm}}^{3}$). When applied to patient data from two clinics, this approach validates the use of a commercial immobilization system for cranial precision radiotherapy.

## ACKNOWLEDGMENTS

The authors acknowledge significant contributions by the mould room and CT simulation personnel at the BC Cancer Agency Vancouver Centre and at the Nova Scotia Cancer Centre. The authors are also thankful to Mr. Ian Porter at the Nova Scotia Cancer Centre for assisting with the experimental apparatus.
